# Primidone: a clinically promising candidate for the treatment of psoriasis

**DOI:** 10.1038/s41420-025-02552-3

**Published:** 2025-06-10

**Authors:** Theresa Riebeling, Benedikt Kolbrink, Rebecca Philippsen, Eileen Dahlke, Tom Delanghe, Franziska Theilig, Agatha Schwarz, Roland Schmitt, Mathieu J. M. Bertrand, Ulrich Kunzendorf, Stefan Krautwald

**Affiliations:** 1 Department of Nephrology and Hypertension, University Hospital Schleswig-Holstein, Kiel, Germany; 2 Department of Dermatology, University Hospital Schleswig-Holstein, Kiel, Germany; 3 Institute of Anatomy, Christian-Albrechts-University Kiel, Kiel, Germany; 4 VIB Center for Inflammation Research, Ghent, Belgium; 5 Department of Biomedical Molecular Biology, Ghent University, Ghent, Belgium

**Keywords:** Biologics, Skin diseases

## Abstract

Using clinically relevant animal models, we have recently demonstrated that the anticonvulsant primidone (Liskantin^®^), approved by the FDA for the treatment of various forms of epilepsy, can effectively block RIPK1 enzymatic activity, which mediates cell death, and consequently prevent RIPK1 cytotoxicity and associated inflammatory responses. Based on these findings, we now reveal both a preventive and, more importantly, a therapeutic effect of primidone in the imiquimod (IMQ)-induced psoriasis-like inflammation model. Notably, the protective effect of IMQ in this necroinflammatory disease is directly correlated with inhibition of the activated state of RIPK1 (as monitored by auto-phosphorylation on Ser166/T169), a critical marker that had been missing in the highly contradictory studies that have previously been published. This allows us to unequivocally identify RIPK1 as a therapeutic target in the treatment of inflammatory disorders, including psoriasis. Given that newly developed RIPK1 inhibitors have shown very limited success in clinical trials for inflammatory and neurological diseases in recent years, and that none of these inhibitors has yet reached clinical utilization, our data strongly recommend a clinical study to evaluate the already approved drug primidone for the treatment of patients suffering from psoriasis within the context of drug repurposing.

## Introduction

Psoriasis, which typically occurs in episodes, is a chronic, inflammatory, noncommunicable skin disease that often requires lifelong treatment, posing a significant medical challenge. Treatment can be highly complex, depending on disease severity, affected body areas, and individual patient responses [1]. Due to the chronic nature of the disease, characterized by a dysregulated immune response, long-term treatments are necessary, which can be costly and burdensome for patients.

In recent years, we and several other research groups have provided compelling evidence through animal studies that a variety of inflammatory diseases, including sepsis, systemic inflammatory response syndrome (SIRS), and various forms of acute kidney injury (AKI), are driven by the receptor-interacting serine/threonine-protein kinase RIPK1 [2–4]. Alongside RIPK3 and the cell death executor MLKL (mixed lineage kinase domain-like protein), RIPK1 is considered a key molecule driving, among others, necroptosis, a specific form of regulated cell death [5]. Compared to other forms of regulated cell death, necroptosis is highly pro-inflammatory because the loss of cell membrane integrity leads to the release of cytokines and damage-associated molecular patterns (DAMPs), which amplify inflammation and activate the immune system. Although contradictory results regarding RIPK1 expression levels in the progression of psoriasis have been published [6, 7], and despite the absence of direct protein-level evidence until now, there is little doubt that activated (phosphorylated) RIPK1 plays a pro-inflammatory role in the pathogenesis of the disease. Inhibiting RIPK1 to reduce excessive inflammatory responses and associated cell damage could therefore represent an innovative and potentially superior therapeutic approach to the existing treatment options for psoriasis. Various small-molecule inhibitors of RIPK1 that offer high selectivity have been developed by different companies and have successfully entered phase I/II clinical trials in humans (source: ClinicalTrials.gov). Few of them are currently being explored as promising therapeutic options for inflammatory diseases such as psoriasis, Crohn’s disease, and rheumatoid arthritis [8]. However, so far, no pharmacological inhibitor of RIPK1-mediated cell death has met the stringent FDA requirements and received approval for clinical use [9]. The most promising candidate in this class has been GSK2982772, but a recently published experimental study using this RIPK1 inhibitor in patients with moderate to severe plaque psoriasis once again yielded only minimal clinical improvements [10]. Although biologics have significantly improved psoriasis treatment in recent years, small molecules continue to face scientific and regulatory challenges that no one has yet managed to overcome. This gap highlights both the complexity of necroinflammatory disease processes and the difficulties in developing an effective and safe alternative, a challenge of great importance for both patients and researchers.

Herein, we aim to demonstrate that the anticonvulsant primidone (Liskantin^®^), already approved for the treatment of various forms of epilepsy, could represent a truly innovative alternative to long-term corticosteroid therapy for chronic inflammatory skin diseases, based on our current findings.

## Results

**Fig. 1:**
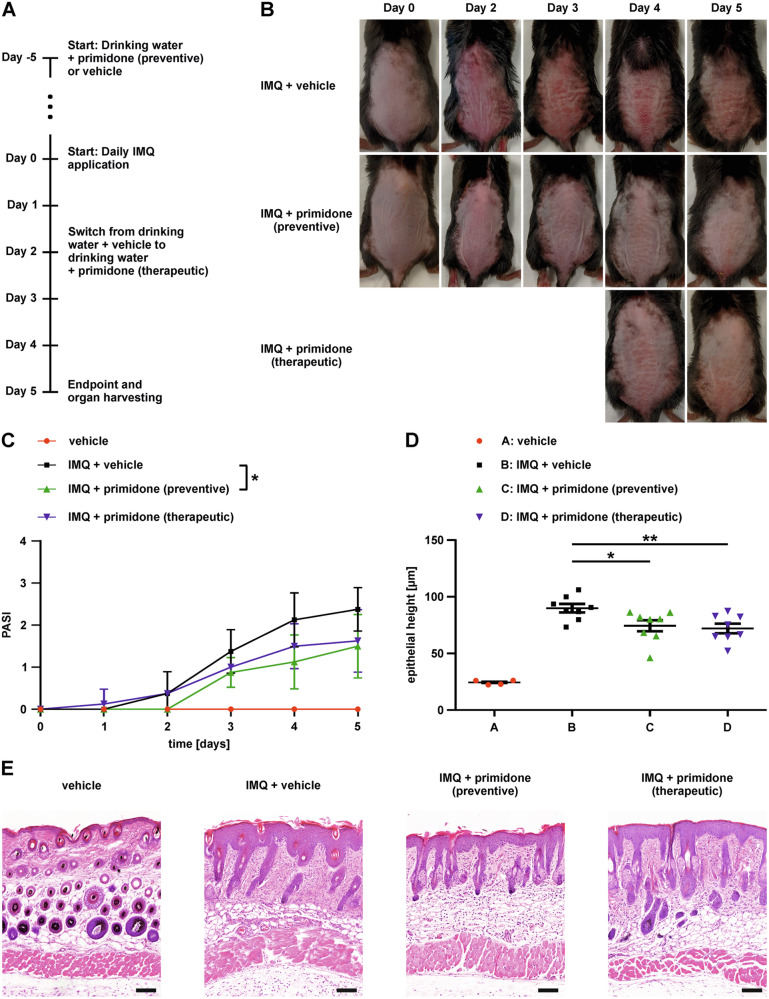
Primidone attenuates clinical symptoms of IMQ-induced skin inflammation. **A** Schematic diagram of the experimental design. Mice received drinking solution containing either 2.875 mM primidone (*n* = 8) or an equivalent amount of dimethyl sulfoxide as a vehicle control (*n* = 4) in their regular drinking water starting five days prior to IMQ application. 62.5 mg of a 5% IMQ-containing cream (Aldara^®^) was applied daily onto the clean-shaven back of 8-week-old female C57BL/6JRj mice while continuously receiving vehicle or primidone via daily prepared drinking water. Two days after the first IMQ treatment, animals of the therapeutic cohort (*n* = 8) were switched from drinking water with vehicle to drinking water with primidone. Five days after the first IMQ application, the animals were sacrificed. **B** Representative images of backs with inflamed skin of mice treated as described in (**A**) over the course of the experiment. **C** The PASI scores were assessed by daily visual examination of all mice. Shown are the mean PASI scores for each day and cohort. Error bars represent mean ± standard deviation (*p < 0.05). **D** The epidermal thickness of the dorsal skin on the 5th day was measured by ten randomly selected fields of two representative sections of each mouse. The mean epidermal height of each animal is depicted as individual data points. Bars show the mean epithelial height of each cohort with standard error (*p < 0.05, **p < 0.01). **E** Microscopic views of H&E staining of representative cross-sectional slices of the dorsal skin of a mouse of each cohort on the 5th day (scale bars represent 100 μm).

To assess the effectiveness of primidone in the course of psoriasis-like inflammation, we employed a murine model in which skin inflammation was induced by daily repeated application of imiquimod (IMQ) to the previously shaved dorsal skin of the animals (model timeline shown in Fig. 1A). To evaluate the fundamental efficacy of primidone in this approach, the substance was administered via drinking water five days before the first IMQ application. Another group of mice received primidone for the first time when primary signs of inflammation, such as skin redness and scaling, became visible (in our case, after two days of IMQ treatment). This approach was taken to assess the therapeutic potential of primidone during psoriasis treatment. The curative effect of primidone in both cohorts is evident in Fig. 1B. The significantly reduced symptoms of skin inflammation under primidone treatment are also reflected in the daily psoriasis area and severity index (PASI) score assessments (Fig. 1C) and the measured epithelial thickness of affected skin regions (Fig. 1D). Histological examinations of skin samples collected at the end of the observation period revealed significant thickening and elongation of the rete ridges in the epidermal layer, which were significantly reduced in both the preventive and therapeutic treatment groups (Fig. 1E).

**Fig. 2:**
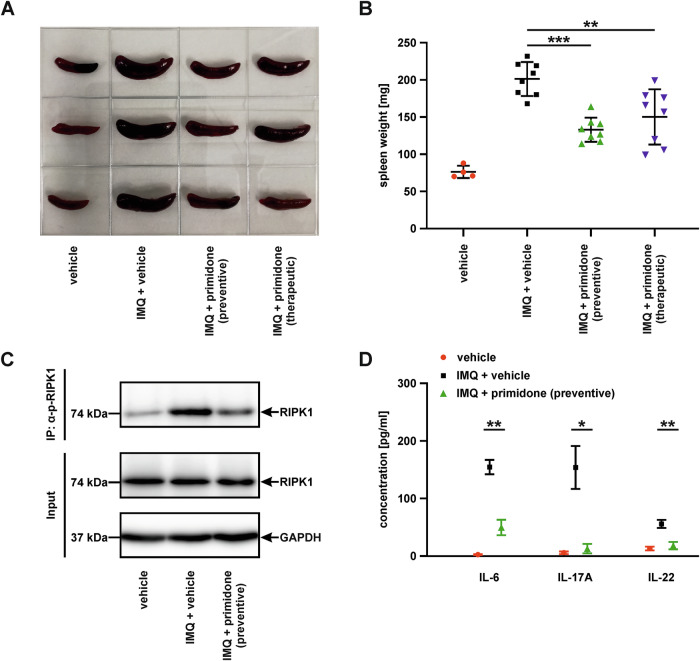
Primidone blocks IMQ-induced splenomegaly, inhibits RIPK1 activation (phosphorylation) at the molecular level, and suppresses the associated inflammatory responses. **A** Representative image of spleens collected after 5 days of IMQ treatment. **B** Quantification of spleen weight following 5 days of IMQ treatment. The weight of each spleen is depicted as an individual data point, bars indicate the mean spleen weight for each cohort with standard deviation (**p < 0.01, ***p < 0.001). **C** Expression levels of the indicated proteins and the activation status (phosphorylation) of the necrosome member RIPK1 in splenocytes from mice treated as shown for 2 days, analyzed by immunoprecipitation and western blotting using specific antibodies (GAPDH served as a loading control). **D** Plasma levels of the indicated inflammatory cytokines were measured two days after the initial application of IMQ-containing cream, both in the presence and absence of primidone, using specific ELISA kits. Data points represent means from three animals per cohort; samples were assayed in triplicate. Data are presented as mean ± standard error (*p < 0.05, **p < 0.01).

Since splenocytes contribute indirectly to psoriasis pathogenesis through their immunoregulatory function and involvement in the production of pro-inflammatory cytokines, the murine IMQ model is known to induce a pronounced splenomegaly [11]. Examination of spleens collected at the end of IMQ treatment showed a marked reduction in organ size (Fig. 2A), which was reflected in a significantly lower spleen weight in mice treated with IMQ + primidone (preventive, as well as therapeutic) compared to the IMQ + vehicle group (Fig. 2B). As we have previously reported that primidone can block RIPK1 kinase activity in the context of necroptosis and inflammation on a molecular level [3], we aimed to verify RIPK1 involvement also in this model. Since RIPK1 activation is an early event in RIPK1-dependent inflammation and cell death signaling, we specifically analyzed the activating phosphorylation of RIPK1 in protein extracts from spleens collected two days after the initiation of IMQ treatment by using a custom-made polyclonal anti-pSer166/T169 RIPK1 antibody [12]. Having information at the post-translational level in tissue samples is often sophisticated, but the specific activation (phosphorylation) of RIPK1 during psoriasis progression, as well as the inhibitory effect of primidone in this setting, is clearly demonstrated in the western blot analysis shown in Fig. 2C, aligning with the previously described experimental results.

Toll-like receptor (TLR) agonizts (agonists instead of agonizts), such as IMQ, are potent immunostimulatory agents. To demonstrate that primidone-mediated inhibition of RIPK1 kinase activity also suppresses the associated inflammatory responses, we measured the levels of proinflammatory cytokines secreted by macrophages and dendritic cells (DCs), cells known to contribute to the pathogenesis of psoriasis [13], two days after initiation of IMQ treatment. The characteristic changes in plasma cytokine levels induced by the disease, as well as the strong therapeutic potential of primidone in this context, are illustrated in Fig. 2D.

## Discussion

In a previous study, we extensively investigated and described the precise mechanism of action of the antiepileptic drug primidone (Liskantin^®^) as a potent inhibitor of RIPK1 kinase activity. In that study, we first identified RIPK1 activation in the airway epithelium of hospitalized patients who tested positive for SARS-CoV-2 and furthermore demonstrated that primidone effectively blocks RIPK1-dependent apoptosis (RDA), necroptosis, and hyperinflammation [3].

In clinical research, drug repurposing is a fascinating strategy as it utilizes existing medications for entirely new therapeutic applications. Since repositioned drugs have already been tested and approved for previously defined conditions, much of the preclinical research phase is bypassed, accelerating development. Repurposed drugs can therefore be used in patients much more quickly, which is particularly beneficial for diseases lacking effective and well-tolerated treatments. RIPK1 is a key regulator of cell fate: its activation determines whether a cell survives or undergoes cell death in response to intracellular stress. Therefore, inhibiting RIPK1 kinase activity may help prevent excessive cell death and subsequent tissue damage, which could be particularly beneficial in diseases characterized by severe inflammation. Our current findings, particularly the direct involvement of activated (phosphorylated) RIPK1 in the examined disease model, support the hypothesis that primidone could be employed to suppress hyperinflammatory processes in clinical practice.

It is well known that a substantial number of approved medications are available for both symptomatic and systemic therapy of psoriasis, positively influencing disease progression [14]. Interleukin-6 (IL-6) is thought to play a central role in the development of psoriasis, as this cytokine can be detected both in the plasma and in the affected skin areas of patients [15]. In addition, other proinflammatory cytokines also contribute to the pathogenesis of the disease. For instance, IL-22 promotes the hyperproliferation and incomplete differentiation of keratinocytes, which leads to the formation of the characteristic plaques seen in psoriasis [16]. However, current therapies often achieve only incomplete remission, lose efficacy over time, or cause unfavorable side effects, highlighting the need for additional treatment strategies. For several reasons, our approach appears both innovative and promising. Current psoriasis therapies primarily focus on modulating the immune system by targeting TNFα, IL-17/IL-23, and PDE-4 inhibitors [17]. Since RIPK1 is the key regulator of necroinflammation [18], its targeted inhibition in psoriasis treatment could introduce a novel therapeutic strategy that simultaneously addresses both cell survival and inflammatory processes. Selective RIPK1 inhibition may help control excessive inflammatory responses in psoriasis without inducing the profound immunosuppression commonly associated with existing biologics and immunosuppressants. Additionally, targeted RIPK1 inhibition could not only modulate immune responses but also reduce skin cell damage, ultimately promoting improved skin regeneration and benefiting patients [19]. Furthermore, some psoriasis patients either do not respond adequately to existing therapies or develop resistance over time. A RIPK1 inhibitor such as primidone could provide a novel treatment option for patients with inadequate responses to current therapies or those who have developed resistance. Finally, to achieve synergistic effects, RIPK1 inhibitors could be combined with existing medications, potentially reducing required dosages and minimizing adverse effects of biologic treatments. In this context, the development of a drug specifically inhibiting the kinase function of RIPK1 represents an innovative molecular approach that has not yet been satisfactorily addressed. It should be noted that a direct comparison between GSK2982772 and primidone in plaque psoriasis is not feasible due to differences in development stages and pharmaceutical dosage forms. Given the well-documented safety, tolerability, pharmacokinetics, and pharmacodynamics of primidone (Liskantin^®^), as well as its approval for epilepsy treatment even in children, it is readily available for clinical application in diseases driven by pathological RIPK1 activation. Thus, primidone could offer a groundbreaking alternative to long-term corticosteroid therapy for chronic inflammatory skin diseases.

## Materials and methods

### Mice

Female C57BL/6JRj mice were obtained from Janvier Labs (Saint Berthevin, France), housed in the Central Animal Facility of the University Hospital Schleswig-Holstein (Kiel, Germany), received standard chow and water *ad libitum* and were held at a 12-h day-night cycle. Mice were 8-weeks-old at the beginning of the experiment.

### Disease model

To induce psoriasis-like inflammation in mice, an area of about 10 cm² of their back was clean-shaven one day prior to the first application of IMQ. Animals were treated daily with 62.5 mg of a 5% IMQ-containing cream (Aldara^®^, MEDA AB, Solna, Sweden) onto their clean-shaven backs to induce skin inflammation. This procedure was carried out until the end of the experiment. Mice were provided with a drinking solution containing either 2.875 mM primidone (Merck Millipore GmbH, Darmstadt, Germany) or an equivalent amount of dimethyl sulfoxide (DMSO) as vehicle control in their regular drinking water. The concentration of primidone we selected for this model is based on the fact that exactly this concentration was evaluated as highly effective and well-tolerated in other in vivo studies that have already been conducted [3].

Clinical symptoms were evaluated according to the PASI score, which was assessed daily as a combined score of the severity of erythema, scaling, and thickening as described previously [20]. To visualize the progression of skin lesions, a trend line was generated based on the mean scores within each experimental group. PASI scores ranged from 0 (no symptoms): no visible erythema or scaling; the skin lesion appeared indistinguishable from healthy skin, over 1 (mild): partial coverage of the lesion with fine scales; the lesion appeared slightly elevated with mild erythema, 2 (moderate): the majority of the lesion was partially or fully covered with scales; moderate fissuring was present, and the lesion exhibited a red, rounded or irregular margin and 3 (severe): nearly the entire lesion was covered with thick, layered scales; pronounced thickening, deep erythema, and distinct fissures were observed to 4 (very severe): complete coverage of the lesion with very thick, layered scales; the lesion was markedly thickened, intensely erythematous, and exhibited prominent fissuring. At the end of the experiment, mice were sacrificed, and skin samples and spleens were collected for further analysis.

### Histology

Freshly collected skin samples were fixed in 4.5% neutral-buffered formaldehyde and embedded in paraffin. Blocks were sectioned to 5 µM thickness and sections were dewaxed, hydrated and subjected to hematoxylin and eosin staining according to routine protocols, dehydrated and finally mounted using DePeX mounting medium (Serva, via Merck Millipore). Epidermal thickening was evaluated in a blinded manner using images captured with a Keyence BZ-x800e microscope (Keyence Deutschland GmbH, Neu-Isenburg, Germany).

### Immunoprecipitation assay

Whole organ protein lysates were obtained from snap-frozen spleen tissue. Tissue samples were mechanically disrupted and lysed in ice-cold IP buffer (50 mM Tris-HCl (pH 7.4), 150 mM NaCl, 1% NP-40, 0.25%, Na-deoxycholate, 1% Triton X-100, 1 mM EDTA, 100 μM Na_3_VO_4_, 1 mM PMSF). Insoluble material was removed by centrifugation (14,000×*g*, 10 min, 4 °C), and the total supernatant of cleared lysates was adjusted to equal volume and protein content and incubated overnight with a custom-made polyclonal anti-pSer166/T169 RIPK1 antibody produced by ThermoFisher Scientific following the 2-rabbit 90-day protocol [12]. Specific pulldown of antibody-bound phosphorylated RIPK1 was performed with μMACS™ Protein G MicroBeads and μ Columns (Miltenyi Biotec GmbH, Bergisch Gladbach, Germany) according to the manufacturer’s instructions. Elution was performed with 40 µl 95 °C SDS loading buffer. For analysis via western blotting, samples were resolved by reducing SDS-PAGE and transferred to a polyvinylidene fluoride membrane (Carl Roth, Karlsruhe, Germany). After overnight incubation with the primary antibody (anti-RIPK1, #3493 from Cell Signaling Technology, Frankfurt, Germany), membranes were washed and probed with secondary antibody (VeriBlot detection reagent (ab131366, Abcam) or Anti-Rabbit IgG (H + L) (111-035-003, Jackson ImmunoResearch). Immune complexes were detected by enhanced chemiluminescence using an ImageQuant 800 detector (Cytiva, Freiburg, Germany). RIPK1 input blot was redeveloped with an antibody against GAPDH (#2118 from Cell Signaling Technology) as a loading control.

### Enzyme-linked immunosorbent assay (ELISA)

To monitor whether primidone-mediated inhibition of RIPK1 kinase activity also suppresses the associated inflammatory responses induced by IMQ, we analyzed the expression levels of multiple inflammatory cytokines in mouse plasma by using specific ELISA kits according to the manufacturer’s instructions. ELISA MAX™ Deluxe Sets specific to mouse IL-6 (#431304), IL-17A (#432504), and IL-22 (#436304) were obtained from BioLegend^®^, Amsterdam, The Netherlands. Absorbance measurements at 450 nm as a specific measure and 570 nm as a reference were performed using a microplate reader (TriStar²S LB 942, Berthold Technologies, Bad Wildbad, Germany).

### Statistical data analysis

Plotting graphs and statistical analysis were performed with GraphPad Prism (version 9) software. Two-tailed Student’s *t*-test was used to determine statistical significance. Significance is indicated as *p < 0.05, **p < 0.01, ***p < 0.001.

## Supplementary information

Supplemental material

## Data Availability

The data generated during the current study are available from the corresponding author upon reasonable request.
