# Supplementary material for: Primidone: a clinically promising candidate for the treatment of psoriasis
Source: Cell Death Discov. 2025 Jun 10;11:275. doi: 10.1038/s41420-025-02552-3 (PMC12152145; doi:10.1038/s41420-025-02552-3)

Fig. 2C

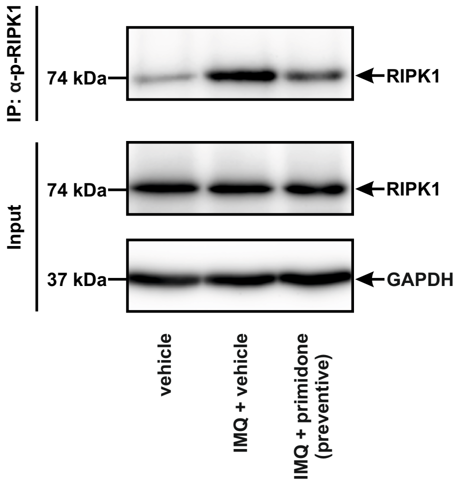

Raw Data (Figure 2C)

Raw Data Luminescence

Overlay with Marker

RIPK1: IP anti p-RIPK1

1. Marker
2. IMQ + vehicle d3
3. IMQ + vehicle d4
4. IMQ + vehicle d5
5. vehicle d2
6. IMQ + vehicle d2
7. IMQ + primidone d2 (preventive)
8. Marker

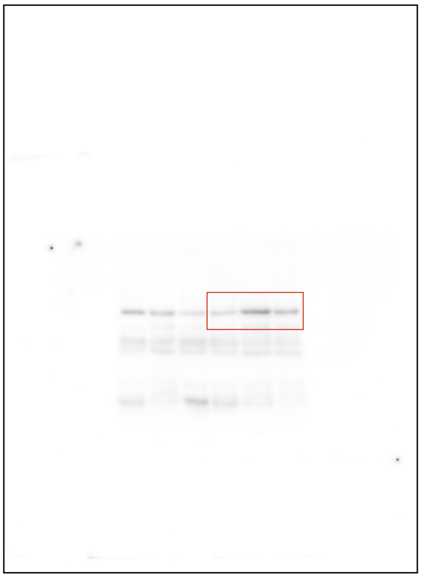

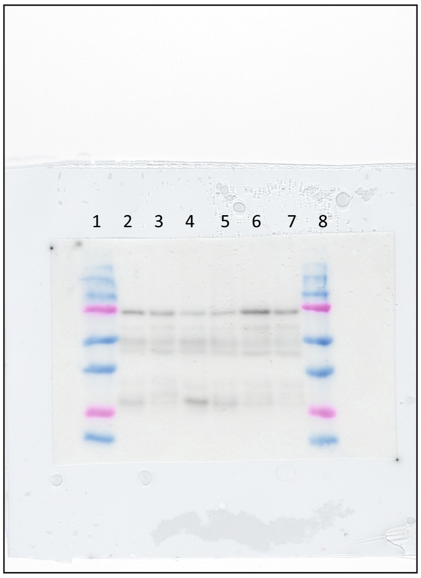

Raw Data (Figure 2C)

Raw Data Luminescence

Overlay with Marker

RIPK1 (input)

1. Marker
2. IMQ + vehicle d3
3. IMQ + vehicle d4
4. IMQ + vehicle d5
5. vehicle d2
6. IMQ + vehicle d2
7. IMQ + primidone d2 (preventive)
8. Marker

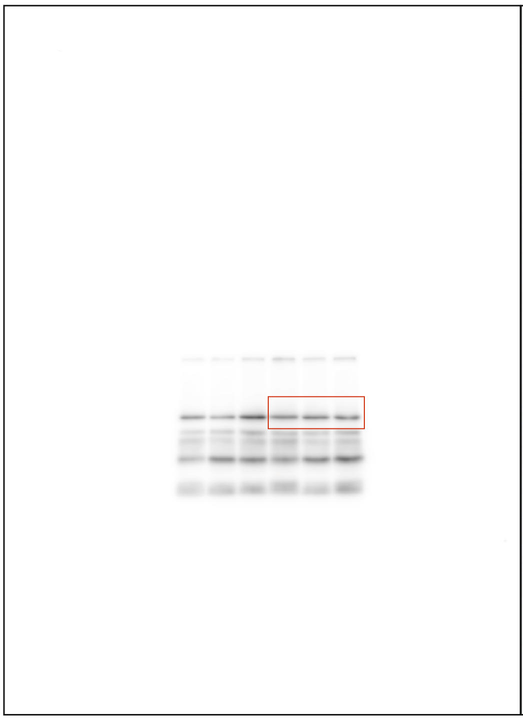

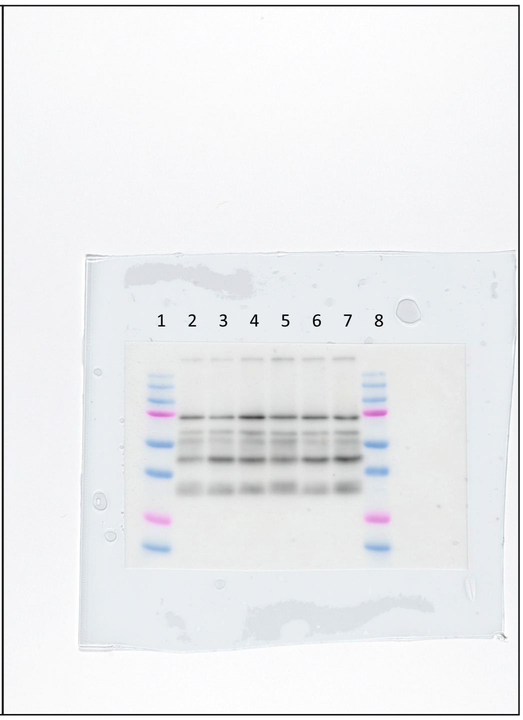

Raw Data Luminescence

Overlay with Marker

GAPDH (input)

1. Marker
2. IMQ + vehicle d3
3. IMQ + vehicle d4
4. IMQ + vehicle d5
5. vehicle d2
6. IMQ + vehicle d2
7. IMQ + primidone d2 (preventive)
8. Marker

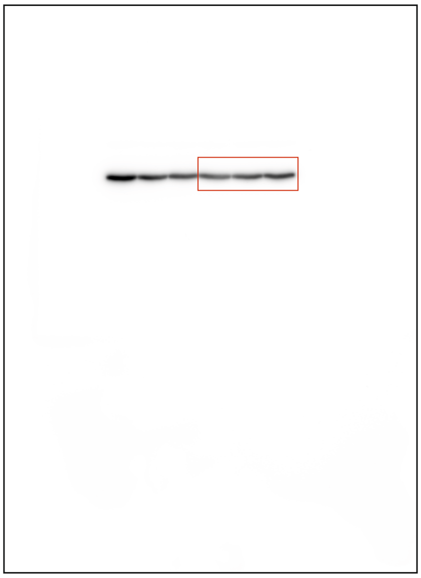

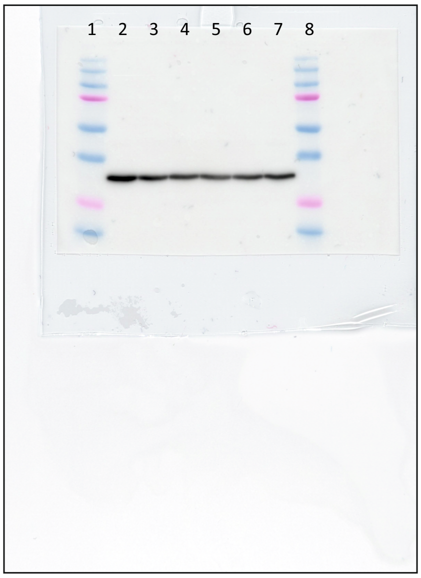

Supplement: Supplementary file 1 — Supplemental material [file 41420_2025_2552_MOESM1_ESM.pdf]
